# A Rare Case of Mediastinal Hydatidosis With Cardiac and Vascular Compression

**DOI:** 10.7759/cureus.83483

**Published:** 2025-05-04

**Authors:** Boujemaa Razouq, Mouhsin Ibba, Walid El Harich, Hicham Fenane, Yassine Msougar

**Affiliations:** 1 Thoracic Surgery Department, University Hospital Mohammed VI, Marrakech, MAR; 2 Thoracic Surgery Department, Cadi Ayyad University, Marrakech, MAR

**Keywords:** echinococcus, helminths, hydatid cyst, mediastinal, thoracotomy

## Abstract

Hydatid disease is a parasitic zoonosis caused by Echinococcus granulosus, typically transmitted through contact with infected dogs or ingestion of contaminated food. Although the liver and lungs are the most commonly affected organs, mediastinal localization is extremely rare and presents diagnostic and therapeutic challenges. This report describes the case of a 48-year-old man with a history of hepatic and pulmonary hydatid disease who developed a retrocardiac mediastinal hydatid cyst (HC). The patient presented with cough, dyspnea, and back pain. Imaging (computed tomography (CT) and magnetic resonance imaging (MRI)) revealed a 9.3 × 7.0 cm cyst compressing mediastinal structures, including the atria and aorta. Surgical removal via posterolateral thoracotomy was performed successfully, and the patient recovered without complications. Albendazole was administered to the patient over a six-month period as part of the postoperative antiparasitic treatment. Echinococcus granulosus causes HCs, a significant zoonotic and pulmonary parasitic disease that can mimic various pathologies and is often harder to manage than the disease itself. HC is considered a significant health problem in India, Iran, China, and Mediterranean countries, which lack satisfactory environmental health, preventive medicine, and veterinarian services. Echinococcosis continues to be a major community health burden in several countries, and in some terrains, it constitutes an emerging and re-emerging disease. Cystic echinococcosis is the most common human disease of this genus, and it accounts for a significant number of cases worldwide. Imaging plays a key role in diagnosis and surgical planning, while surgery remains the mainstay of treatment, supported by medical therapy.

## Introduction

Hydatidosis, also referred to as hydatid cyst (HC), is a global zoonosis caused by the larval stage of Echinococcus granulosus, whose primary host is stray dogs. Humans become infected by ingesting contaminated food or by contact with infected dogs [1]. HC can impact nearly any organ in the body, with symptoms varying depending on the affected organ and the disease stage. While it primarily affects the liver and lungs, HC can also involve other areas like the pleural cavity, mediastinum, and diaphragm. Once ingested, the parasite spreads through the bloodstream to different organs. The liver and lungs act as filters, limiting the spread of the parasite, which is why HC is rare in other organs [2,3]. Mediastinal localization of HCs is exceedingly rare and presents significant diagnostic and therapeutic challenges [4]. This report presents a case of mediastinal localization of HCs.

## Case presentation

**Figure 1:**
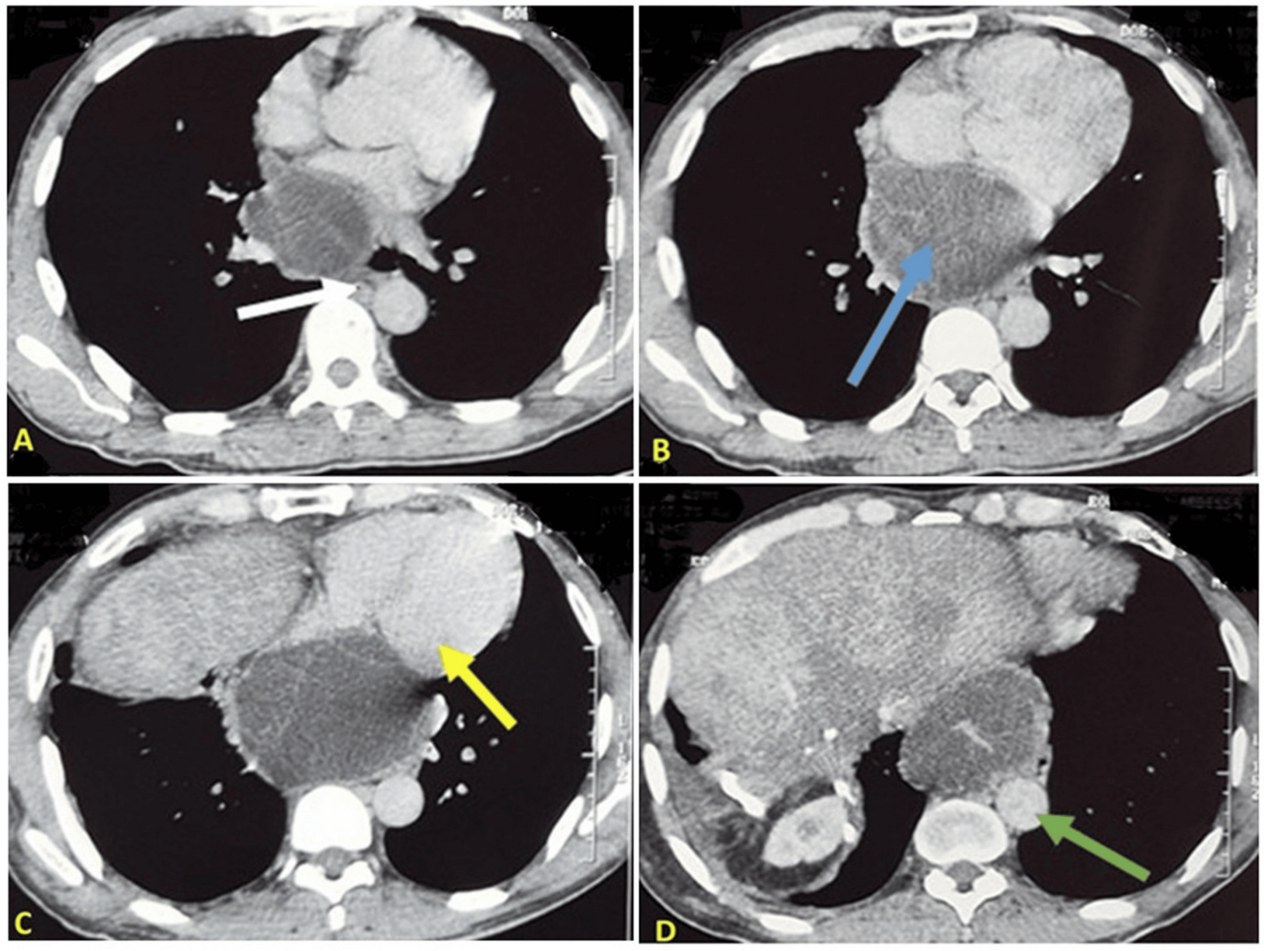
Thoracic CT scan: Image B shows a well-defined hydatid cyst located in the inferior retrocardiac mediastinum (blue arrow), measuring 9.3 × 7.0 cm. The lesion displaces and compresses adjacent mediastinal structures, including the atria (Image C, yellow arrow), thoracic aorta (Image D, green arrow), and esophagus (Image A, white arrow).

A 48-year-old male with a two-year history of diabetes mellitus managed with metformin, and a one-year history of hypertension treated with amlodipine 5 mg, presented for evaluation. The patient, originally from a rural area with dog exposure, has a history of hepatic and pulmonary hydatid surgeries performed 28 and 21 years ago, respectively. Two months prior to presentation, he experienced Sadoul Stage 2 dyspnea, classified by a scale that grades dyspnea severity based on physical activity, along with a cough producing whitish sputum (without hemoptysis), back pain, and a sensation of right-sided thoracic heaviness. These symptoms occurred in the context of afebrile status and stable general health. Clinical examination yielded no significant findings. A chest X-ray revealed an inferior mediastinal enlargement. Subsequent computed tomography (CT) imaging demonstrated a fluid accumulation in the inferior mediastinum, located superior to the diaphragm. The fluid formation was oval in shape, measuring 9.3 x 7.0 cm, and exhibited no enhancement following the administration of contrast medium. The formation exhibited well-defined contours and a thin, regular wall containing logettes separated by septa. It was in close proximity to the atria, causing lateral compression of the left great azygos vein, descending aorta, and esophagus, all of which were displaced to the left. Post-contrast injection revealed discrete enhancement of the wall and septa. The absence of intraparenchymal HC was noted (Figure 1).

**Figure 2:**
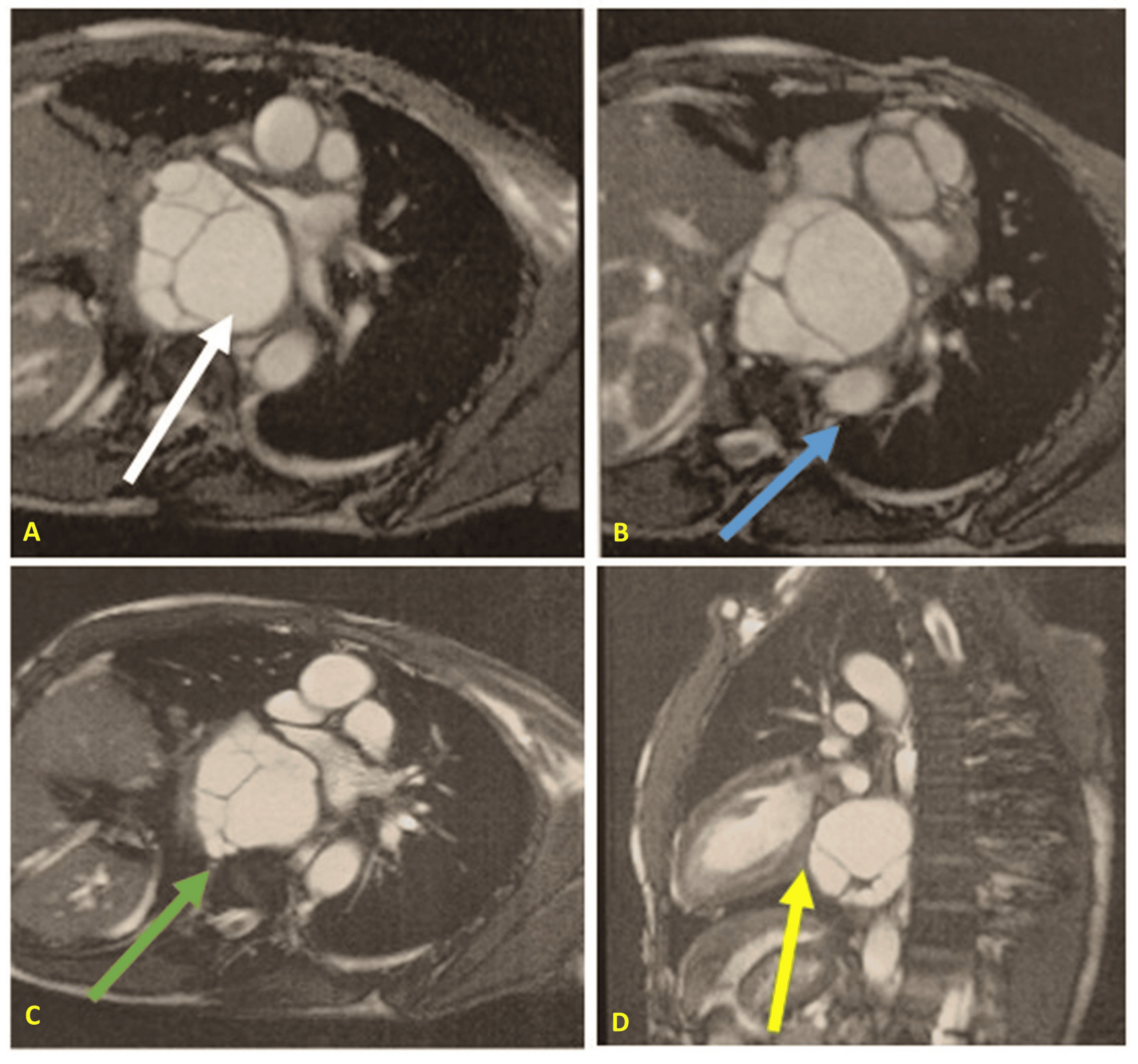
Magnetic resonance imaging shows a cyst located in the posterior-inferior mediastinum (Image A, white arrow), in close contact with the spine but without evidence of spinal cord invasion (Image C, green arrow). The cyst compresses the atria anteriorly without signs of infiltration (Image D, yellow arrow) and displaces the aorta to the left (Image B, blue arrow).

An abdominal CT scan revealed a dysmorphic liver, which is consistent with postoperative changes, and no suspicious lesions were identified. Due to the close proximity to the atria, cardiac magnetic resonance imaging (MRI) was performed, demonstrating compression of the atria without evidence of invasion (Figure 2).

Laboratory results showed hyperleukocytosis with 13,930 cells/mm³ (reference range: 4,500-11,000 cells/mm³), and the serological test, performed by indirect hemagglutination, was positive. Other tests, including the intradermal (Casoni) test, complement fixation (CF), bentonite flocculation (BFI), latex agglutination (LA), and indirect immunofluorescence assay (IFA), were not performed in this case.

**Figure 3:**
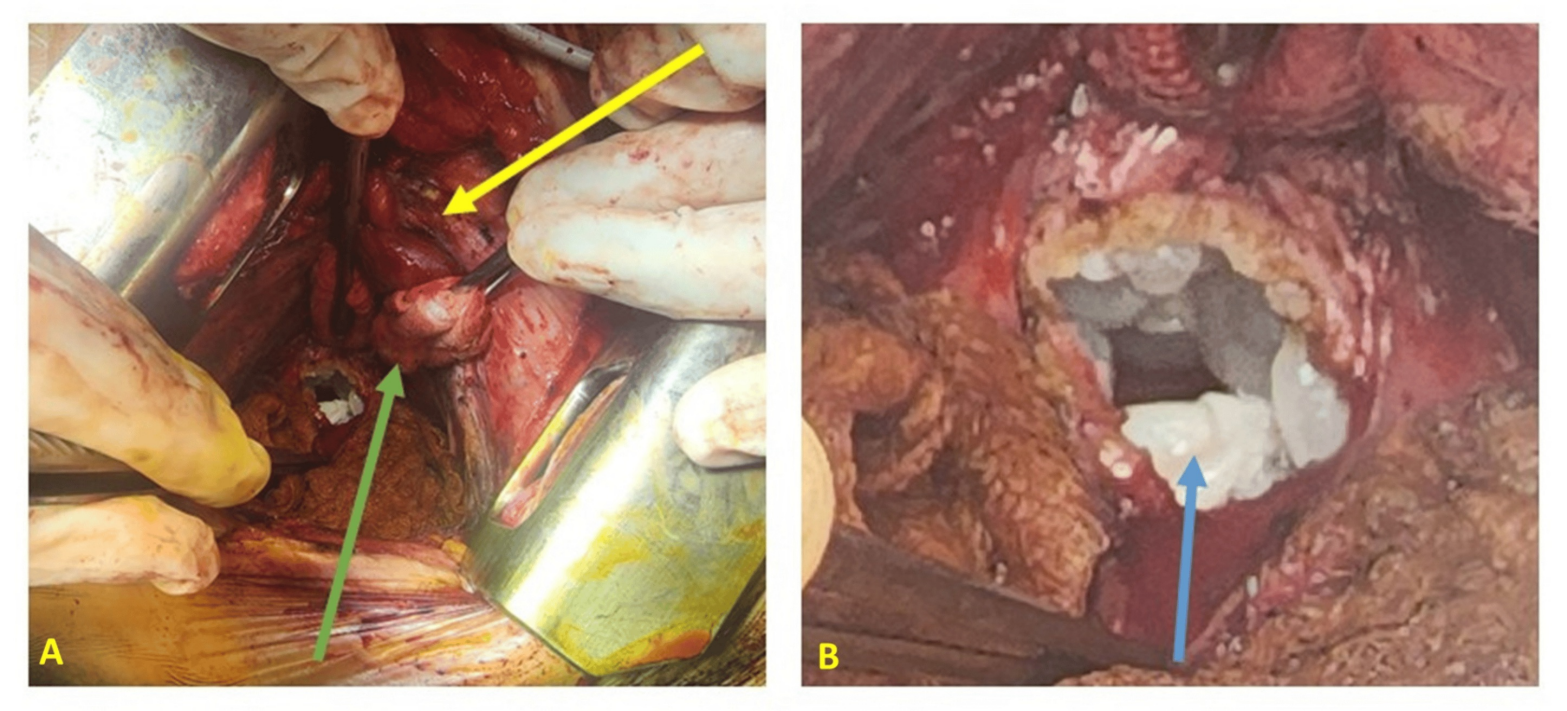
Intraoperative images: Image A shows the lung retracted anteriorly (yellow arrow) and the atria protected by a mounted pad (green arrow). Image B displays the pericyst and cyst membrane (white), along with daughter vesicles (blue arrow).

The patient underwent a posterolateral thoracotomy through the sixth right intercostal space, revealing a posterior retrocardiac mediastinal cystic mass that was compressing the atria posteriorly. Following the protection of the surgical field with compresses soaked in betadine and hypertonic saline solution, the mediastinal pleura was meticulously incised opposite the mass, situated between the azygos vein posteriorly and the atria anteriorly, thereby exposing the white hydatid membranes. Subsequent needle aspiration of the cyst facilitated the removal of several vesicles (Figure 3).

**Figure 4:**
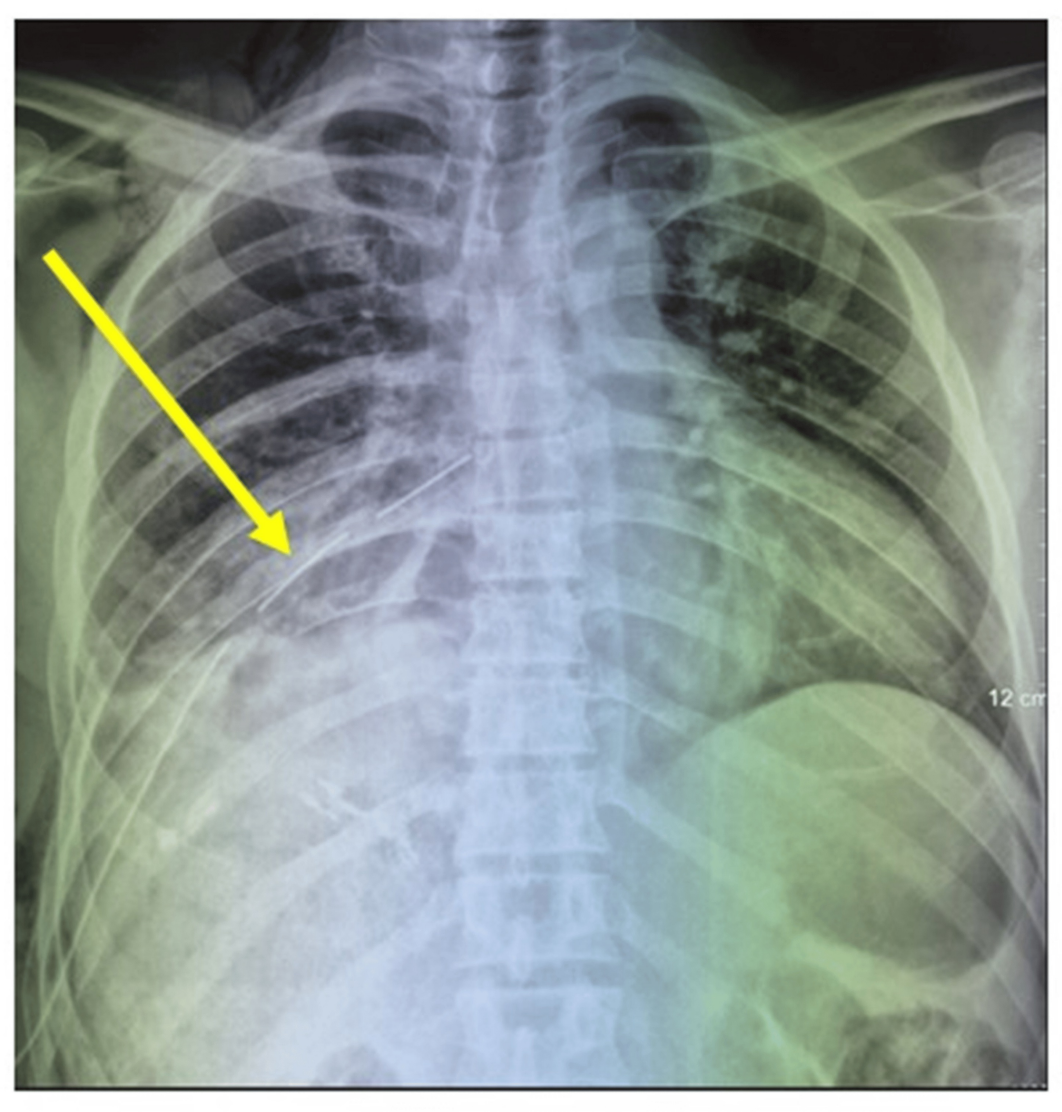
Chest X-ray revealed a lung adherent to the wall, with a drain in place (yellow arrow) and disappearance of mediastinal opacity.

The mediastinal cavity was extensively irrigated with hydrogen peroxide. The postoperative course was deemed satisfactory, demonstrating positive clinical, biological, and radiological progression (Figure 4).

The patient received postoperative treatment with albendazole for six months, at a dose of 400 mg (one tablet daily), with favorable progress and no recurrence.

## Discussion

Mediastinal hydatid cyst (MHC) is relatively rare, accounting for around 0.5-2.6% of cases of hydatid disease [3,4]. Primary localization in the mediastinum is one of the rarest manifestations of the condition, with around 100 cases documented in the English literature [4]. The mechanism of occurrence in the mediastinum can be either direct dissemination via the systemic circulation or rupture of a peripheral pulmonary cyst. In addition, cysts may spread through the diaphragm or abdominal lymphatic system [5]. In the present case, given the patient's history of hepatic and pulmonary hydatidosis, it is highly likely that the mediastinal localization resulted from intraoperative contamination from the pulmonary cyst or from primary systemic seeding.

MHC can cause symptoms such as chest pain, cough, dyspnea, dysphagia, and dysphonia, depending on the size of the cyst, its location, and the involvement of neighboring structures. The diagnosis may be made incidentally or following complications, such as rupture of the cyst into a major vessel or into the heart [1]. In our case, the patient's persistent cough, dyspnea, and back pain were caused by compression of neighboring structures such as the atria, large vessels, and esophagus.

Imaging is essential for the diagnosis and staging of MHC. Thoracic radiography guides the diagnosis with rounded or oval opacities of watery tone. Thoracic ultrasound confirms the diagnosis, revealing the fluid nature and proliferative membrane characteristic of HCs. CT of the thorax confirms the diagnosis, showing a mass of fluid density, often with contrast in the pericyst and peripheral calcifications. HC lesions often occur in the posterior mediastinal compartment [6,7]. Mediastinal MRI is used in cases where patients cannot tolerate iodinated contrast media or during pregnancy. Together with ultrasound and CT, it can differentiate MHC from other cystic masses. In uncertain cases, the diagnosis is confirmed during surgery [3]. HCs can be difficult to distinguish from benign or malignant tumors due to their varying size and shape. MRI helps in characterizing the cyst and its contents, making it easier to differentiate HC from other cystic lesions in the affected organ [8]. In our case, CT was insufficient to rule out invasion of the atria and esophagus, which justified the use of MRI. The initial diagnosis was of hydatid origin given the history of pulmonary and hepatic hydatidosis, but the operative risk was pejorative given the close relationships with the atria and aorta.

Hydatid serology is a key tool for preoperative diagnosis, though a negative result does not exclude the condition [9]. In our case, serology was positive, and given the endemic context, the patient’s history of hepatic and pulmonary hydatidosis, and supportive imaging findings, the diagnosis of HC is highly likely.

Surgical treatment is crucial for MHC and typically involves cystectomy combined with either total or partial pericystectomy. Total pericystectomy is rarely feasible due to the close proximity of the cyst to vital structures, making partial pericystectomy the more common approach. This consideration guided our decision in the present case, where a partial pericystectomy was performed. The choice of surgical approach depends on the specific case and may include posterolateral thoracotomy, anterolateral thoracotomy, or median sternotomy [6,9]. The postoperative recovery is usually straightforward, with no reported mortality or recurrence [3]. In our patient, due to the close relationship between the cyst, the atria, and the major vessels, the pericyst was carefully opened, considering the risk of hemorrhagic complications due to the compression. A cystectomy with capitonnage (Barrett's method) was performed without any significant perioperative or postoperative complications. The various surgical options for pulmonary HCs are given in Table 1 [10].

**Table 1:** Various surgical procedures for pulmonary hydatid disease.

Surgical approach	Description
Enucleation (Ugon method)	The Ugon enucleation technique (1952) involves the complete surgical removal of the hydatid cyst from the lung parenchyma, with an alternative method using positive pressure ventilation to remove the cyst spontaneously, leaving the pericyst behind.
Pericystectomy (Perez-Fontana method)	The Perez-Fontana method of pericystectomy, proposed in 1953, involves the excision of the hydatid cyst along with the pericyst, which adheres firmly to the normal lung parenchyma. Closure of the airway openings and approximation of the healthy lung parenchyma are essential.
Cystostomy with capitonnage (Barrett’s method)	Proposed by Barrett in 1952, this method involves cystostomy followed by capitonnage of the residual cavity. It has largely remained the treatment of choice for conservative approaches, with most prevalent techniques being based on Barrett’s method.
Capitonnage and bronchial tube closure following cystectomy (Posadas method)	A contemporary of Barrett, Posadas proposed a modified version of Barrett’s procedure, in which the bronchial openings were closed prior to performing capitonnage.
Cystotomy with closure of bronchial openings alone	This technique, recently introduced by Turna, Erdogan, and Eren et al., is a modification of the Posadas method that omits the capitonnage step.
Figuera’s open aspiration technique	Figuera’s technique is analogous to the PAIR method used for hepatic hydatidosis and involves the aspiration of cyst membranes and daughter cysts.
Segmental resection	Liaras et al. performed the first segmental resection for a hydatid cyst in 1955, using a conventional resection technique similar to that employed for other pulmonary conditions.
Lobectomy	Pulmonary lobectomy was initially performed in 1950 to treat an inflammatory lesion of the lung.
PAIR technique	The procedure is carried out under CT or ultrasonographic guidance, following all necessary precautions and with emergency medications readily available.

Albendazole is the preferred medical therapy for hydatidosis, offering better efficacy and absorption than mebendazole. However, its role in the definitive treatment of the disease remains debated [11].

## Conclusions

In summary, although rare, HC should be considered in the differential diagnosis of mediastinal cystic lesions especially in endemic regions and in patients undergoing surgery for hepatic or pulmonary HCs. CT scan of the thorax is the most efficient method of diagnosing these lesions. Surgical resection was successful in all cases and remains the treatment of choice for MHC; additional adjuvant medical therapy is essential to avoid recurrence. The case presented here highlights the diagnostic and therapeutic challenges posed by MHCs, especially when located in close proximity to the atria and aorta.
